# Sex differences in protein expression in the mouse brain and their perturbations in a model of Down syndrome

**DOI:** 10.1186/s13293-015-0043-9

**Published:** 2015-11-09

**Authors:** Aaron Block, Md. Mahiuddin Ahmed, A. Ranjitha Dhanasekaran, Suhong Tong, Katheleen J. Gardiner

**Affiliations:** Department of Pediatrics, Linda Crnic Institute for Down Syndrome, Aurora, USA; Colorado School of Public Health, Aurora, USA; Human Medical Genetics and Genomics, and Neuroscience Programs, University of Colorado Denver School of Medicine, 12700 E 19th Avenue, Mail Stop 8608, Aurora, CO 80045 USA

**Keywords:** Trisomy 21, Dp(10)1Yey, Mouse chromosome 10, Hippocampus, Learning and memory deficits, TRPM2, S100B, Cerebellum, Intellectual disability

## Abstract

**Background:**

While many sex differences in structure and function of the mammalian brain have been described, the molecular correlates of these differences are not broadly known. Also unknown is how sex differences at the protein level are perturbed by mutations that lead to intellectual disability (ID). Down syndrome (DS) is the most common genetic cause of ID and is due to trisomy of human chromosome 21 (Hsa21) and the resulting increased expression of Hsa21-encoded genes. The Dp(10)1Yey mouse model (Dp10) of DS is trisomic for orthologs of 39 Hsa21 protein-coding genes that map to mouse chromosome 10 (Mmu10), including four genes with known sex differences in functional properties. How these genes contribute to the DS cognitive phenotype is not known.

**Methods:**

Using reverse phase protein arrays, levels of ~100 proteins/protein modifications were measured in the hippocampus, cerebellum, and cortex of female and male controls and their trisomic Dp10 littermates. Proteins were chosen for their known roles in learning/memory and synaptic plasticity and include components of the MAPK, MTOR, and apoptosis pathways, immediate early genes, and subunits of ionotropic glutamate receptors. Protein levels were compared between genotypes, sexes, and brain regions using a three-level mixed effects model and the Benjamini-Hochberg correction for multiple testing.

**Results:**

In control mice, levels of approximately one half of the proteins differ significantly between females and males in at least one brain region; in the hippocampus alone, levels of 40 % of the proteins are significantly higher in females. Trisomy of the Mmu10 segment differentially affects female and male profiles, perturbing protein levels most in the cerebellum of female Dp10 and most in the hippocampus of male Dp10. Cortex is minimally affected by sex and genotype. Diverse pathways and processes are implicated in both sex and genotype differences.

**Conclusions:**

The extensive sex differences in control mice in levels of proteins involved in learning/memory illustrate the molecular complexity underlying sex differences in normal neurological processes. The sex-specific abnormalities in the Dp10 suggest the possibility of sex-specific phenotypic features in DS and reinforce the need to use female as well as male mice, in particular in preclinical evaluations of drug responses.

**Electronic supplementary material:**

The online version of this article (doi:10.1186/s13293-015-0043-9) contains supplementary material, which is available to authorized users.

## Background

Sex differences in brain function and dysfunction are well documented [1, 2]. Sex differences in learning strategies, in responses to stress, and in the effects of stress on learning have been described in both rodents and humans; possibly contributing to these are differences in adult neurogenesis that have been described in rodents [2–4]. Neuropsychiatric disorders, such as schizophrenia, major depression, and post-traumatic stress disorder, show sex biases in incidence, age of onset, and/or severity [5]. Neurodegenerative diseases also show sex differences; notably, Alzheimer’s disease (AD) is more common in women than men [6]. Sex differences are also seen in efficacy and side effects of drugs used to treat such disorders [7].

Sex differences are attributed at least in part to molecular events that occur during development and throughout postnatal life in the regulation and levels of sex hormones and their receptors [8, 9]. Sex differences also exist in expression of some genes encoded by the X chromosome. As many as 15 % of human X chromosome genes have been reported to escape silencing on the inactive X, which may result in higher levels of expression in females of these X inactivation escape genes [10–14]. Because sex hormone receptors and X inactivation escape genes together include transcription factors and genes involved in post-translational protein modifications, effects of sex differences will propagate downstream to affect many pathways and cellular processes. Indeed, in one comprehensive study where oligonucleotide arrays were screened with RNA from >100 age-matched female and male mice, of the ~4500 genes with detectable expression in brain, ~600 (14 %) showed significant differences in levels between sexes; ~350 were higher in females and ~260 were higher in males [15].

### Intellectual disability and Down syndrome

Intellectual disability (ID) affects 1–3 % of the population worldwide [16]. For ID associated with genetic causes, mutations in several hundred human genes have been identified [17, 18]. Of these, ~100 are encoded by the X chromosome, which contributes to the elevated incidence of ID in males [19]. The most common genetic cause of ID, however, is Down syndrome (DS), with an incidence of 1 in ~700–1000 live births worldwide [20, 21]. DS is caused by trisomy of all or part of the long arm of human chromosome 21 (Hsa21q) and the increased expression of trisomic genes. Hsa21q encodes ~160 proteins of diverse functions, ~50 members of the keratin-associated protein family, multiple microRNAs, and several hundred human-specific transcripts that may be protein coding, functional RNAs, or transcriptional noise [22, 23]. The neurological phenotype of DS includes well-documented cognitive deficits in tasks requiring a functioning hippocampus, executive function, and language processing [24–26]. Neuronal numbers and cellular morphology are abnormal in several brain regions, including the hippocampus and cerebellum. Of particular importance, now that the life span of people with DS has increased to >60 years, is the universal development of the pathology of AD by age 30 and the development in half of those with DS of an AD-like dementia by the age of 50 [27]. While much has been determined about the functions of some Hsa21 genes, the true number and identity of all those contributing to ID and AD in DS is not known. Sex differences in the specifics of ID in DS, and whether these simply reflect sex differences in the typical population, have not commonly been investigated.

### Modeling DS in mouse and the Dp(10)1Yey

DS is difficult to model well in mouse, not only because it is a contiguous gene syndrome with candidate genes spanning the length of Hsa21q but also because orthologs of Hsa21 genes are distributed on segments of three mouse chromosomes: the telomeric region of mouse chromosome 16 (Mmu16) and internal segments of mouse chromosome 17 (Mmu17) and mouse chromosome 10 (Mmu10) [22]. Many partial trisomy mouse models of DS have been created, and each shows a unique constellation of DS-relevant learning deficits, synaptic plasticity and cellular abnormalities, and/or gene expression perturbations [28]. Little, however, has been determined regarding sex differences. Here, we focus on the Dp(10)1Yey mouse model of DS (abbreviated Dp10). The Dp10 was generated by chromosomal engineering to carry an internal duplication spanning the 39 Hsa21 orthologs mapping to Mmu10 [29]. Several of these genes have been shown individually to have roles in brain development and function, to modulate molecular processes involved in AD, and/or to display sex differences in their functional properties and consequences. The following genes are examples: (i) the adenosine deaminase 2 (ADAR2) gene encodes a protein that modifies the activities of glutamate, gamma-aminobutyric acid (GABA), and serotonin receptors by means of pre-mRNA editing [30–33]; a null mutation of ADAR2 results in deficits in the auditory startle response and hearing impairment in male, but not female, mice [34]; (ii) the Ca-binding protein, S100B, stimulates neurite outgrowth and the activation of microglia and, when overexpressed, exacerbates AD-like pathology in a mouse model of AD [35, 36]; (iii) the cysteine protease inhibitor, cystatin B, CSTB, when mutated causes a form of progressive myoclonic epilepsy [37, 38] and, when knocked down, rescues AD-like features in a mouse model of AD; (iv) the collagen 18A1 C-terminal fragment, endostatin, functions in synaptogenesis in the cerebellum [39] and, at least in vitro, can inhibit neurite outgrowth and neuronal migration [40]; (v) the protein methytransferase, PRMT2, and (vi) the small ubiquitin-like modifier protein, SUMO3, methylate and sumoylate, respectively, steroid hormone receptors;—both modifications contribute to the regulation of activity of estrogen, androgen, progesterone, and other receptors that function during development and in the adult brain [41–43]; and (vii) the transient receptor potential cation channel, subfamily M, member 2,TRPM2, contributes to N-methyl-D-aspartate (NMDA)-mediated metaplasticity in hippocampal synapses [44]; a null mutation of TRPM2 protects male, but not female, mice from damage due to ischemia with a mechanism that involves the androgen receptor [45]. Because orthologs of these genes map to Hsa21, it is reasonable to predict that overexpression in DS would impact levels and/or activities of many non-Hsa21 proteins, and consequently, brain structure and function, and that it would do so with direct or indirect sex differences.

Overexpression at the RNA level of ADAR2, S100B, and TRPM2 has been demonstrated in the Dp10 mice [29]. This was not, however, associated with deficits in learning and memory (LM) when mice 2–4 months old were tested in the Morris water maze (MWM) and context fear conditioning (CFC), nor with abnormal long-term potentiation (LTP) [46]. In contrast to behavioral and electrophysiological data, when levels of 26 non-Hsa21proteins relevant to AD were measured in the hippocampus of ~8-month-old Dp10 mice, levels of 12 differed significantly from those in controls [47]. Only male mice were reported in both these studies.

To investigate sex differences in molecular features that may underlie normal LM and perturbations that may contribute to impaired LM in DS, we describe here expression levels of ~100 proteins in the hippocampus, cortex, and cerebellum of cohorts of male and female control mice and their age- and sex-matched trisomic littermates from the Dp10 line. The proteins include components of the MAPK, MTOR, and apoptosis signaling pathways, immediate early gene (IEG) proteins, subunits of ionotropic glutamate receptors, and additional proteins involved in synaptic plasticity and/or known to be mutated in subsets of patients with ID or in mouse mutants showing LM deficits or abnormal in patients with AD or mouse models of AD. We show that, in the hippocampus of control mice, levels of almost half of these proteins differ between male and female mice and that in each case, the level is higher in females than in males. In contrast, sex influences on levels of the same proteins are minimal in the cortex and cerebellum. Trisomy-associated perturbations are also sex-specific, with hippocampus most affected in male Dp10 and cerebellum most affected in female Dp10. The observation that females and males differ in their baseline profiles of proteins critical to learning and memory suggests that molecular responses to the stimulation of learning will differ. The data also suggest that Hsa21 genes with orthologs on Mmu10 may influence, positively or negatively, cognitive features in people with DS, and that the molecular basis of these features, and their modulation by pharmacological treatment, will differ between males and females.

## Methods

### Mice

The Dp(10)1Yey mice [29, 46], originally a gift from Y. Yu (Roswell Park, New York), were maintained by breeding trisomic males to non-trisomic females on a C57BL/6JEi background. The mice were housed at the University of Colorado Denver in a room with HEPA-filtered air and a 14:10 light:dark cycle and fed with 6 % fat diet and acidified (pH 2.5–3.0) water ad libitum. All procedures were approved by the University of Colorado Institutional Animal Care and Use Committee and performed in accordance with the National Institute of Health guidelines for the care and use of animals in research. Seven litters of mice were used, comprising 10 female control mice, 7 female Dp10 trisomic mice, 9 male control mice, and 10 male Dp10 trisomic mice. Littermates (Additional file 1), separated by sex, were housed in the same cage. Female mice (with two exceptions noted) were in diestrus. All mice were naïve, aged 7–9 months, and sacrificed between 12 p.m. and 2 p.m. to maintain a consistent circadian time point.

### Genotyping of mice

DNA was prepared from a 1-mm tail snip by lysis in 50 nM NaOH at 98 °C for 1 h, followed by neutralization with 1 M Tris Base (pH 8.0). Lysates were stored at −20 °C until use. Mice were genotyped by standard PCR using the following primer pairs: control gene reverse primer 5-′CTAGGCCACAGAATTGAAAGATCT- 3′ and forward primer 5′GTAGGTGGAAATTCTAGCATCATCC 3′; trisomic gene forward primer 5′GGCGAACGTGGCGAGAAA 3′ and reverse primer 5′CCTGCTGCCAAGCCATCAG 3′.

### Tissue processing and protein lysate preparations

To preserve protein phosphorylation, mice were sacrificed by cervical dislocation without anesthetic. The whole brain was removed, immediately snap frozen in liquid nitrogen, and stored at −80 °C. For lysate preparation, the brains were removed from the freezer and, without thawing, rapidly heated to 95 °C under vacuum in the Stabilizor T1 (Denator, AB) as described previously (48). The cortex, hippocampus, and cerebellum were dissected out, weighed, placed in 10 volumes of IEF buffer (8 M urea, 4 % CHAPS, 50 mM Tris) and homogenized by sonication with three bursts 5 s long in a Branson Sonic Power Co. (Danbury, CT). Lysates were centrifuged to remove debris, and the protein concentration of the cleared supernatant was determined using the 660 nM Protein Assay Kit (Pierce); all sample protein concentrations were 9–11 mg/ml. Information for each mouse, age, littermates, and tissue weight is provided in Additional file 1. Gonadal hormone levels were not measured.

### Antibodies and validation for RPPA

Proteins screened for expression level are listed in Additional file 2. Functional annotation as ID or LM and antibody information regarding supplier, catalog number, and dilution factor are also provided. Reverse phase protein arrays (RPPA) require highly specific antibodies. Prior to use, each lot of each antibody was verified by Western blot using mouse brain lysates to show only clean band(s) of explainable size, with no non-specific bands present. All secondary antibodies (IgG; anti-goat, rabbit, and mouse) have been shown previously to produce signals that are less than 5 % above local background when incubated with an RPPA slide in the absence of any primary antibody; signals of these levels are too low to be reliably quantitated and were ignored in data analysis.

### Array assembly and printing

Each sample lysate was prepared in 5 dilutions, neat plus 4 serial dilutions with a 0.8 dilution factor, and 1 buffer control, in a 384-well V-shaped ABgene plate (Thermo Fisher Scientific, Rockford, IL). Samples were printed, in triplicate, onto nitrocellulose-coated glass slides (Grace Bio-Laboratories, Inc., Bend, OR) using an Aushon BioSystems 2470 Arrayer (Aushon BioSystems, Billerica, MA) with 185-μm pins and a single touch. The arrays were produced in two major print runs and slides were stored at 4 °C until further use.

### Antibody detection and array staining

Procedures for array screening have been described previously [48]. Briefly, slides were incubated in blocking solution 3 % BSA (Sigma, USA) in TBST (Tris-buffered saline, 0.1 % Tween 20) for 4 h, followed by overnight incubation at 4 °C with shaking with the primary antibody (antibody dilutions are provided in Additional file 2: Table S2). Detection of the bound primary antibody was performed by incubation with the secondary antibody, Fluorescence Alexa Fluor 555 goat anti-mouse or anti-rabbit or rabbit anti-goat (1:2000 dilution) (Invitrogen, Carlsbad, CA), for 90 min at room temperature. Slides were washed and dried, and signals were detected by scanning on a GenePix Pro 4000B array slide scanner (Axon Instruments, USA) using GenePix 4.0 software or on a PerkinElmer Scan Array Express HT Microarray Scanner (PerkinElmer Inc., MA, USA). For normalization, total protein for each spot was determined by staining three non-sequential slides from each print run with SyproRuby reagent (Invitrogen, CA, USA) following the manufacturer’s protocol.

### Image analysis, quantification, normalization, and statistical analysis

Signals on each slide were quantified using Scan Array Express software (PerkinElmer Inc., MA, USA) where the antibody signal intensity for each spot was normalized to the corresponding SyproRuby signal. Details of quantification and review of data quality and reproducibility were as described previously [48, 49, 50]. After removal of technical outliers, normalized protein values, transformed into a natural log scale were used in statistical analysis. Mean differences between genotypes (trisomy vs. control) and sexes (female vs. male) were reported as a ratio and percent, assessed using a hierarchical three-level mixed effects model to account for possible correlations and variability between replicates and dilutions within each sample. The Benjamini-Hochberg corrected *p* value <0.05 with a false discovery rate (FDR) of 5 % was considered for overall statistical significance across the entirety of the hypotheses. Results of all comparisons carried out for the three brain regions are provided in Additional file 3.

For correlation analysis, data were reduced to one observation per mouse. Protein values for each brain region of each individual of each sex/genotype were used to compute Spearman correlation coefficients. Graphs for data from protein pairs with correlation coefficients greater than 0.8 with *p* < 0.05 were inspected and correlations with artifactually high *r* values (i.e., non-linear relationships) were eliminated. All data analysis was carried out using SAS® version 9.3 (SAS Institute Inc., Cary, NC).

### Protein interaction networks

Protein interaction partners of each protein encoded in the Dp10 trisomic segment for each of the proteins measured by RPPA and for proteins encoded on the X chromosome that escape X inactivation [11–13] were obtained from the IntACT (http://www.ebi.ac.uk/intact/), HPRD (Human Protein Reference Database, http://www.hprd.org/), and BioGRID (Biological General Repository for Interaction Datasets, http://thebiogrid.org/) databases. Subsets of primary and secondary interactions for sex hormone receptors and proteins screened by RPPA were retained for networks in Fig. 7. Networks were constructed using Cytoscape 3.0.2.

## Results

The goals of the protein measurements were first to assess sex differences in control mice and then to determine how trisomy of the Hsa21 syntenic region on Mmu10 influences both sex-dependent and sex-independent protein profiles. A total of ~100 proteins/protein modifications were screened in whole tissue lysates from the hippocampus, cortex, and cerebellum of ~8-month-old mice. Four pairwise comparisons were carried out for each brain region: (i) protein levels in control females were compared to those in control males to determine sex differences normally present in the inbred C57BL/6JEi background, (ii) levels in trisomic females were compared to those in trisomic males to determine if and how trisomy alters normal sex differences, (iii) levels in trisomic males were compared to those in control males, and (iv) levels in trisomic females were compared to those in control females, to determine sex-independent and sex-specific perturbations caused by trisomy. Proteins measured included 18 components of the MAP kinase pathway and 14 from the MTOR pathway, 4 immediate early gene proteins, subunits of ionotropic glutamate receptors, and a number of proteins associated with AD. Fourteen proteins encoded by Hsa21, 4 of which are trisomic in the Dp10, were also measured. Proteins were chosen because of their specific individual importance, or the importance of the pathways in which they function, to LM or synaptic plasticity or because they have been shown to be abnormal in brains from people or mouse models of DS, ID, or AD. Proteins include those used in previous studies of the Tc1 mouse model of DS and of the Ts65Dn with and without memantine treatment and exposure to CFC [48, 49]. The complete list of proteins is provided in Additional file 2, which includes annotation as ID or mouse LM proteins. The use of RPPA requires highly specific antibodies, and as a result, some proteins of interest could not be assayed. Results of the measurements of all proteins in the three brain regions in all four comparisons are provided in Additional file 3. In the following, we first summarize the general features of the protein profiles with respect to sex and genotype differences. We then discuss details for specific proteins and pathways affected by sex and genotype.

### Summary of sex differences

**Fig. 1:**
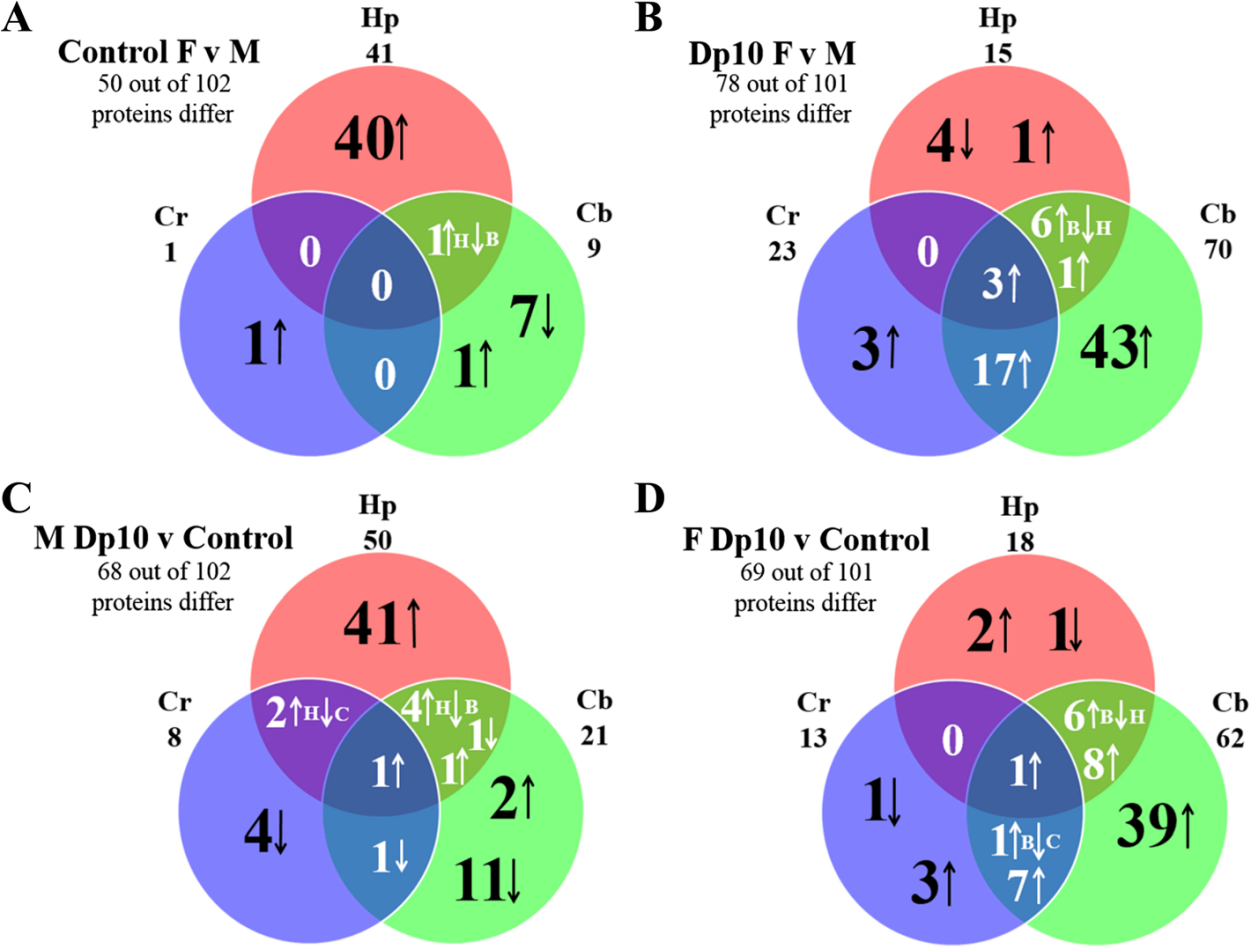
Distribution and overlaps of sex and genotype protein differences among brain regions. The number of proteins showing different levels in at least one brain region and the total number of proteins measured in each comparison are provided in each panel. In each Venn diagram, the total number of proteins that differed is indicated under the name of the brain region. *Pink* hippocampus (*Hp*), *green* cerebellum (*Cb*), *blue* cortex (*Cr*). *Arrows* within the Venn diagram *circles* indicate increases and decreases in the respective ratios. *H* hippocampus, *B* cerebellum, *C* cortex. **a** Female controls vs. male controls. **b** Female Dp10 vs. male Dp10. **c** Male Dp10 vs. male controls. **d** Female Dp10 vs. female controls

Figure 1a shows a Venn diagram illustrating, in control mice, the distribution among brain regions of sex differences in protein expression. Of 102 proteins measured, levels of 50 differed significantly between females and males in at least one brain region. Hippocampus overwhelmingly showed the most differences, 41. In addition, levels of all of them were higher in females than males and, for all but one protein, the differences were specific to the hippocampus. In comparison, sex differences were seen in only nine proteins in the cerebellum and in only one protein in the cortex. A single protein differed in more than one brain region: IL1B differed in both the hippocampus and cerebellum, elevated in females in the former and decreased in the latter.

Figure 1b illustrates the number of brain region sex differences in protein expression in trisomic mice. In contrast to control mice, the hippocampus showed the fewest sex differences, only 15, and the cerebellum showed the most, with 70 proteins differing in levels. Also in contrast to controls, many proteins (a total of 27) showed significant sex differences in more than one brain region. Specifically, only 43 of 70 showed sex differences uniquely in the cerebellum; in both the cerebellum and cortex, levels of 17 proteins were higher in female trisomics than those in males, and three proteins were higher in females in all three brain regions. Levels of seven proteins differed between sexes in both the hippocampus and cerebellum, but for six of these, the differences were in opposite directions: levels were lower in females than those in males in the hippocampus and higher in the cerebellum.

It is evident from Fig. 1a, b that in addition to differences in brain region distribution, trisomic mice show a greater total number of sex differences than do control mice. As shown in Table 1, of ~300 measurements (~100 in each of the three brain regions), a total of 51 measurements (~16 %) showed sex differences in control mice, while 107 measurements (~35 %) differed between sexes in trisomic mice. The magnitudes of the sex differences are also greater in trisomy. In control mice, 27 of the 51 differences were in the range of 15–30 % and only two differed by >30 %. In contrast, in trisomy, a majority of differences in both the hippocampus (9 of 15) and cortex (16 of 23) were in the range of 15–30 %, and in the cerebellum alone 30 of 70 differences were >30 %. In total, in trisomy, 88 of 107 differences were >15 %. Therefore, trisomy not only changes the identity and brain region distribution of the sexually dimorphic proteins but also exacerbates the magnitude of sex differences.

**Table 1:** Distribution among brain regions, direction (female vs. male), and relative magnitude of sex differences

	Hp (100)	Cb (101)	Cr (97)	Total
Controls				
Increased	41	1	1	51
Decreased	0	8	0	
Δ15–30 %	23	4	0	27
Δ > 30 %	0	2	0	2
Trisomy				
Increased	5	69	23	107
Decreased	10	0	0	
Δ15–30 %	9	29	16	54
Δ > 30 %	0	30	4	34

*Hp* hippocampus, *Cb* cerebellum, *Cr* cortex. Δ, difference between sexes of the same genotype. Brackets, total number of proteins measured

### Perturbations due to trisomy

Figure 1c shows that, in the comparison of cohorts of male mice, levels of 68 of 102 proteins were altered due to trisomy. The hippocampus showed the most perturbations, with 50 proteins altered. Of these, all but one protein were increased in trisomy and 41 increases were hippocampus-specific. In the cerebellum, 21 proteins differed from controls, of which 11 were decreased and 2 were increased uniquely in the cerebellum. Few proteins were altered in more than one brain region. Six proteins were affected in both the hippocampus and cerebellum, although levels of four changed in opposite directions. In cortex, only eight proteins were altered, four were decreased uniquely. A single perturbation was common to all three brain regions: levels of the trisomic protein S100B were increased by ~25–30 %. Perturbations in additional Hsa21 orthologs are discussed below.

Figure 1d shows that, in trisomic females, levels of the majority of proteins measured, 69 of 101, differed from controls in at least one brain region. The distribution of perturbations among brain regions, however, differed from that in males. Only 18 perturbations were seen in the hippocampus. Instead, the majority occurred in the cerebellum where a total of 62 proteins were altered. The cortex again was minimally affected, with only 13 proteins altered. Fourteen proteins were perturbed in both the hippocampus and cerebellum, although only eight were altered in the same direction. Eight proteins were altered in both the cerebellum and cortex, and seven of these were changed in the same direction in both regions. As with male mice, S100B was elevated in both the hippocampus and cerebellum (it was not measured in cortex). Also elevated in all three regions was the non-Hsa21 protein AKT.

As shown in Table 2, the magnitudes of the perturbations were greater in female trisomics than those in male trisomics. More than half, 56 (60 %) of the total of 93 perturbations, seen in the three brain regions were in the range of 15–30 % in female trisomics, compared with only 36 (45 %) of the total of 79 perturbations seen in male trisomics. Furthermore, 19 of 93 perturbations were >30 % in females compared with only 6 of 79 in males.

**Table 2:** Distribution among brain regions, direction (trisomic vs. control) and relative magnitude of trisomic differences

	Hp (100)	Cb (101)	Cr (97)	Total
Females				
Increased	11	62	11	93
Decreased	7	0	2	
Δ15–30 %	8	40	8	56
Δ > 30 %	3	14	2	19
Males				
Increased	49	4	1	79
Decreased	1	17	7	
Δ15–30 %	24	10	2	36
Δ > 30 %	4	2	0	6

*Hp* hippocampus, *Cb* cerebellum, *Cr* cortex. Δ, difference between trisomic and control of same sex. Brackets, total number of proteins measured

### Trisomy and sex effects on levels of Hsa21 orthologous proteins

**Fig. 2:**
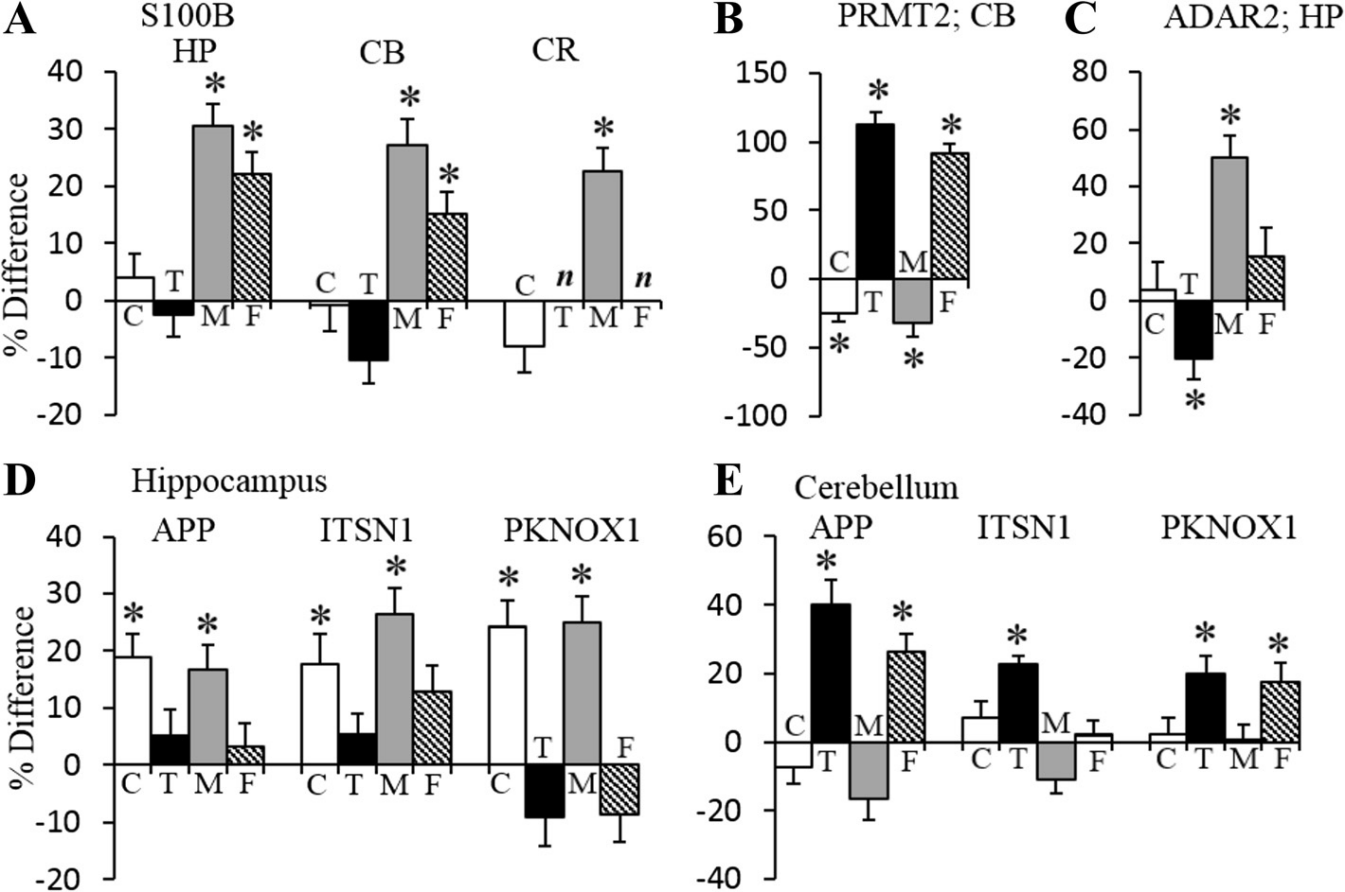
Sex and genotype differences in levels of selected Hsa21-encoded proteins. *Bar graphs* indicate the percent (%) increase or decrease in each comparison. *HP* hippocampus, *CB* cerebellum, *CR* cortex, *C* controls, females vs. males; *T* trisomic (Dp10) females vs. Dp10 males; *M* male Dp10 vs. male controls; *F* female Dp10 vs. female controls. *Asterisk* significant difference by three-level mixed effects model after Benjamini-Hochberg correction, with 5 % false discovery rate. *n*, not measured. *Error bars* indicate the SEM. **a**–**c** Proteins are encoded by genes trisomic in the Dp10 mice. **d**, **e** Genes encoding APP and ITSN1 map to Mmu16 and PKNOX1 maps to Mmu17 and are not trisomic in the Dp10

Levels of 14 Hsa21 orthologs were measured. Genes encoding three, S100B, PRMT2, and ADAR2, are trisomic in the Dp10 mice. As shown in Fig. 2a, levels of S100B are uniformly elevated by 20–30 % in trisomy in all three brain regions and do not differ with sex in controls or trisomics. Levels of PRMT2 were not significantly affected by sex or by trisomy in the hippocampus (Additional file 3), but in the cerebellum, they showed very large perturbations, especially in females (Fig. 2b). In female control mice, PRMT2 cerebellar levels were 25 % lower than those in male controls; however, levels increased by 90 % in trisomic female mice, but decreased by ~30 % in male trisomic mice. This causes a reversal of sex differences in trisomy, where levels in trisomic females are >100 % higher than those in trisomic males. Figure 2c shows that in the hippocampus, ADAR2 was elevated by 50 % in male Dp10 but not significantly affected by trisomy in females. In the cerebellum, ADAR2 was not affected by sex or trisomy (Additional file 3) (ADAR2 was not measured in the cortex).

Proteins encoded by four Hsa21 genes that are not trisomic in the Dp10 were also affected by sex and/or trisomy with brain region specificities (Fig. 2d, e). In the hippocampus of control mice, APP, ITSN1, RCAN1, and PKNOX1 were elevated by 13–24 % in females compared with males. Trisomy of the Mmu10 region resulted in increases in the levels of these proteins in the hippocampus of male mice but had no effect in females. This sex-specific response served to erase sex differences in trisomy. In the cerebellum, however, there were no sex differences in levels of these proteins in control mice; trisomy produced significant changes only in female mice, with the result that levels of APP, ITSN1, and PKNOX1 were significantly higher in female Dp10 than in males (Fig. 2e).

Consistent with the overall modest sex and trisomy effects on protein expression in cortex, perturbation of Hsa21 protein levels were slight. Levels of S100B were elevated in trisomy (PRMT2 and ADAR2 were either unchanged or not measured in all samples). Unique to cortex, however, sex comparisons showed significantly higher levels in Dp10 females than Dp10 males of the Hsa21 orthologs TIAM1, CBS, and RRP1 (Additional file 4). These were largely a result of increases in trisomy females compared to control females, with trisomy not affecting males.

There are two important observations here: levels of some proteins encoded by Hsa21 orthologous genes that are not trisomic are affected by trisomy of the Mmu10 region. These genes include APP that has been well-studied for its role in AD. The effects on these proteins are sex-biased, which means that many would be missed in studying only male cohorts.

### MTOR pathway

**Fig. 3:**
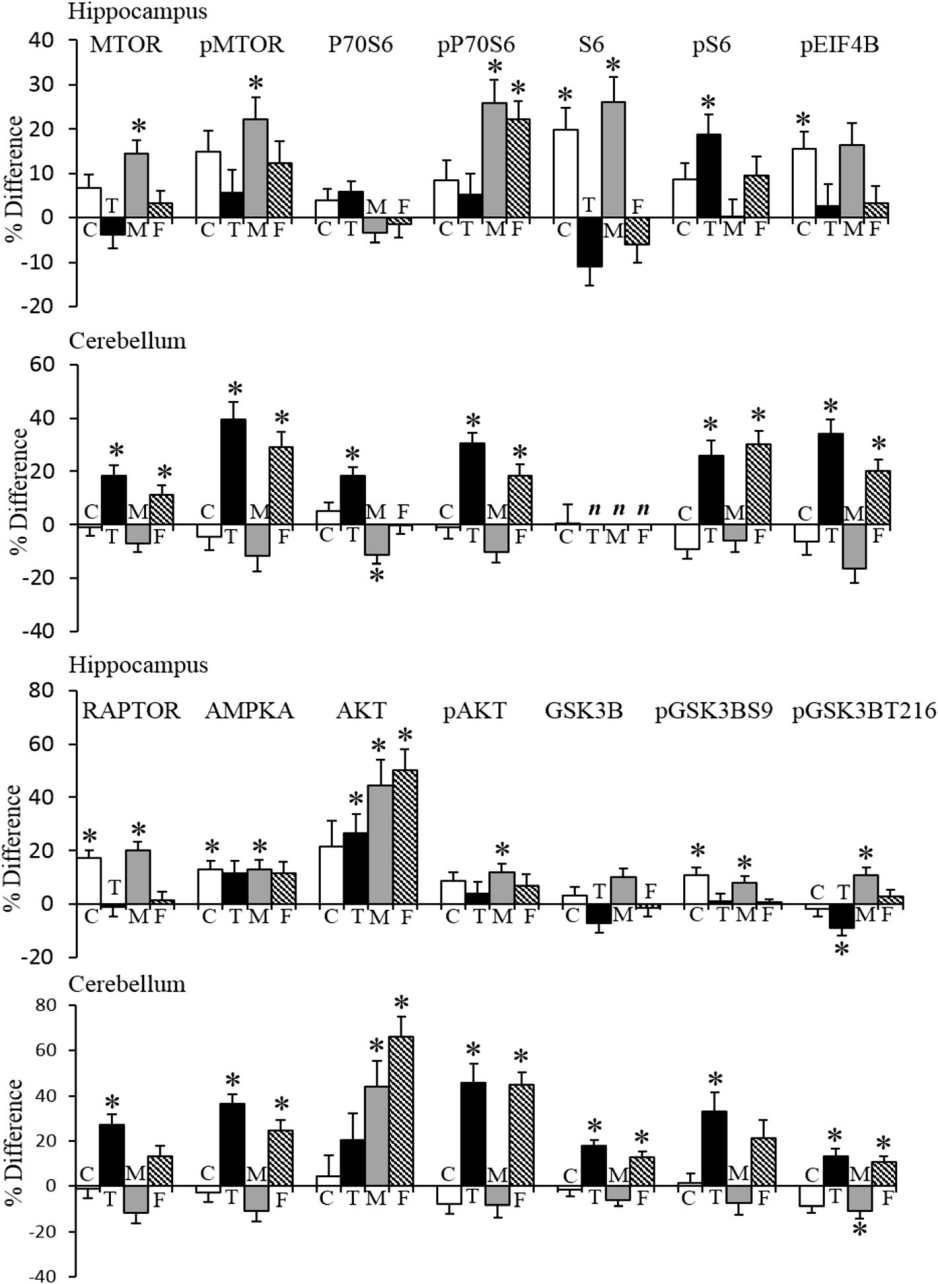
Sex and genotype differences in levels of components of the MTOR pathway in the hippocampus and cerebellum. Legend as in Fig. 2

In the hippocampus, 14 components of the MTOR pathway were measured, including 7 phosphoproteins. Results are shown in Fig. 3. Levels of five components differ between female and male controls, showing differences of 11–20 %. In Dp10 males, levels of 10 of the 14 components were increased with respect to control males; only pEIF4B, GSK3B, P70S6, and pS6 were not affected. The opposite occurred in Dp10 females: levels of 12 of 14 components were *not* altered, and only AKT and pP70S6 were increased with respect to control females. The scenario in the cerebellum is very different. There were no significant sex differences in control mice, and only three proteins, AKT, P70S6, and pGSK3BY216 were altered in male trisomic mice. However, in female trisomic mice, levels of 9 of 13 components (S6 was not measured) were significantly increased; of these, AKT and pAKT were increased by 66 and 45 %, respectively, and pMTOR and pS6 each by ~30 %. As a result, levels of 12 of 13 components of the MTOR pathway were higher in the cerebella of female Dp10 mice than male; only AKT shows similar levels in male and female trisomics. Thus, the picture of perturbations in MTOR is strongly sex, genotype, and brain region-specific.

### MAPK pathway

In comparison to MTOR, perturbations in the MAPK pathway are minimal. In the hippocampus, four components differed between male and female control mice, pERK, pELK, RSK, and pRSK, and only pBRAF and pERK showed sex differences in Dp10 mice. Only 5 of 14 components of the classical MAPK pathway were perturbed, all increased, in male Dp10, and only one was altered in female Dp10. Data are shown in Additional file 4.

### AD-related proteins

We previously reported in the hippocampus of male Dp10 mice [47] measurement of 26 proteins that had shown abnormal levels in brains of patients with AD and/or mouse models of AD. We extended this analysis here to additional brain regions and cohorts of female mice. In control mice, 10 proteins showed sex differences in the hippocampus, all elevated in females (Additional files 3 and 4). Among them were APP, ERBB4, pSRC, IL1B, pNUMB, and CASP3, proteins that were also perturbed in male Dp10. When trisomic females were compared with control females, however, levels of only 4 of the same 26 proteins were abnormal, and only pTau and α-synuclein were perturbed in both male and female Dp10. Levels of four proteins, CDK5, pGSK3BY216, IL1B, and nNOS, showed sex differences in trisomic mice. Data are provided in Additional file 3, and a subset is shown in Additional file 4.

Data from the cerebellum again present a very different picture. Levels of only four of the 26 AD-related proteins, CDK5, IL1B, NR1 and NR2B, differed between sexes in controls, while the majority, 18 of 26, differed between sexes in trisomic mice. Levels of nine proteins were altered in male Dp10, and 16 were altered in female Dp10. Perturbations in the levels of five proteins, CDK5, pGSK3BY216, NR1, P35/25, and pNUMB, were of the same magnitude in both male and female trisomics, but opposite in direction, decreased in males and increased in females relative to their respective controls. These data are shown in Additional file 4.

### Sex and genotype effects in the cortex

**Fig. 4:**
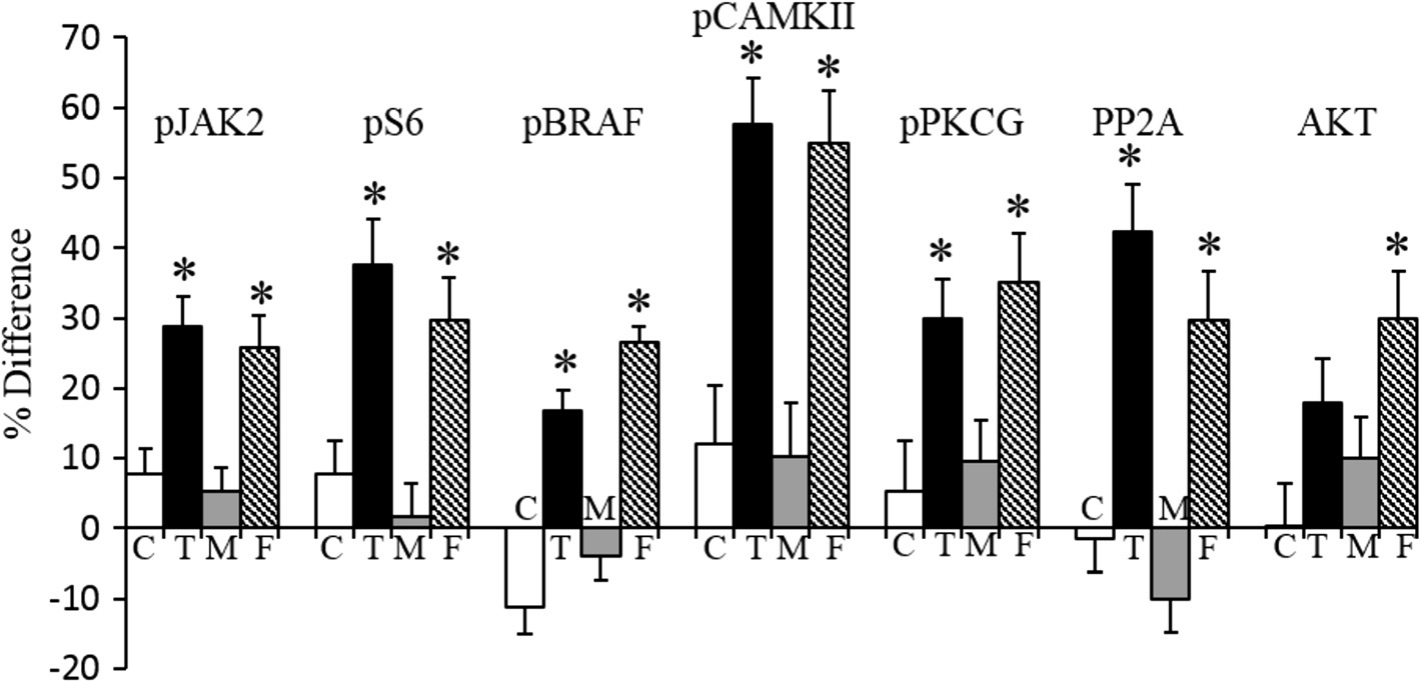
Sex and genotype differences in levels of selected proteins in cortex. Legend as in Fig. 2

Compared with hippocampus and cerebellum, protein levels in the cortex showed few sex differences and genotype perturbations. However, as shown in Fig. 4, the perturbations that did occur affect proteins of particular interest to LM and brain function, and the differences were both unique to females and strong. For example, levels of pCAMKII were increased by 50 % in female Dp10, pS6 and PP2A by 35 %, and pJAK2 and pPKCG, each by 25 %.

### Relationships between brain regions

**Fig. 5:**
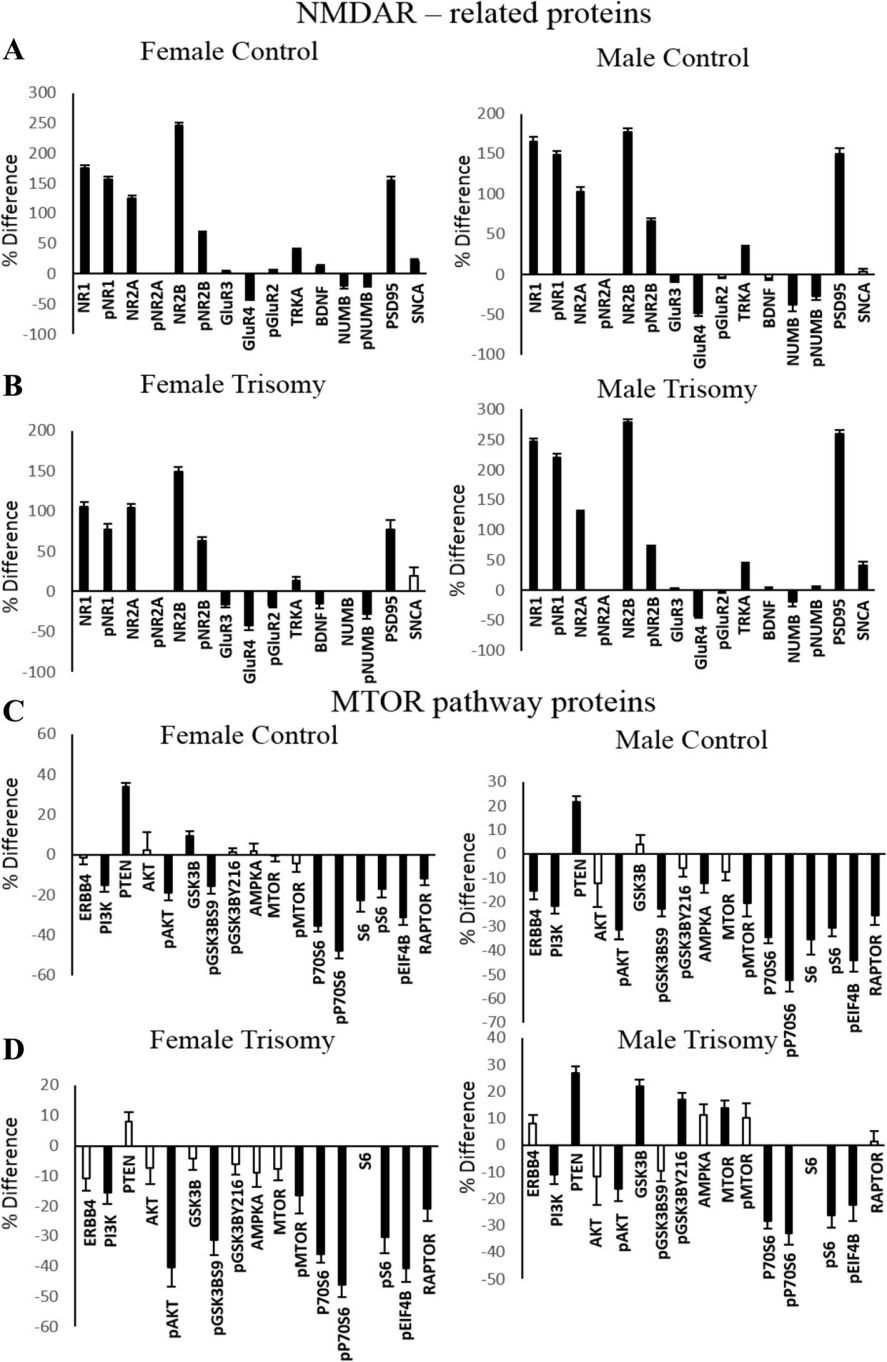
Ratio of protein levels in hippocampus and cerebellum. **a** NMDAR subunits and related proteins in female and male control mice. **b** NMDAR subunits and related proteins in female and male Dp10 mice. **c** Components of the MTOR pathway in female and male control mice. **d** Components of the MTOR pathway in female and male Dp10 mice. *Y axis* % difference in hippocampus vs. cerebellum. *Black bars* significant difference, *white bars* non-significant difference

We compared protein levels between brain regions in controls and asked how these relationships changed, or not, with trisomy. The bar graphs in Fig. 5 show the significant differences between the hippocampus and cerebellum in the levels of NMDAR subunits and the components of the MTOR pathway. A positive ratio indicates higher levels in the hippocampus, and a negative ratio indicates higher levels in the cerebellum.

Figure 5a shows data for NMDAR subunits and related proteins in female and male control mice. Overall, the patterns are very similar. With the exception of pNR2A, levels of all NMDAR subunits, plus TRKA and PSD95, are significantly greater in the hippocampus, and by similar magnitudes, in both females and males. These relationships are preserved even when protein levels differ between sexes, e.g., for NR1 and NR2B. Similarly, levels of GLUR4, NUMB, and pNUMB are higher in the cerebellum than those in the hippocampus, in both females and males. For only three proteins, GLUR3, BDNF, and SNCA, the higher levels in female hippocampus vs. those in male hippocampus result in modest differences in the hippocampus and cerebellum levels between sexes.

Figure 5b shows results of the same analysis in trisomic mice. For both female and male Dp10, the patterns of proteins that are higher in the hippocampus and higher in the cerebellum are similar to those in their sex-matched controls. Notably, however, in female Dp10, the magnitudes of the differences are smaller, e.g., levels of NR1 and pNR1 are 100 and ~75 % higher in the hippocampus than those in the cerebellum in trisomic females but ~170 and ~150 % higher in the hippocampus in controls. This reflects the predominant perturbation of protein levels specific to the cerebellum in trisomic females and the relatively few perturbations in hippocampus. Conversely, in male Dp10 mice, the levels of differences between brain regions are generally higher than those in male controls, e.g., levels of NR2B and PSD95 are ~250 % higher in the hippocampus than those in the cerebellum in male Dp10, but only ~170 and ~150 % higher in male controls. This in turn reflects the effects of trisomy in male Dp10 on protein levels in the hippocampus.

Figure 5c, d present a similar analysis for components of the MTOR pathway. For both female and male control mice (Fig. 5c), the differences in protein levels between the hippocampus and cerebellum are smaller than those for NMDAR subunits and levels of the majority of proteins are lower in the hippocampus than those in the cerebellum. There is, however, an overall similarity in patterns. In particular, levels of PI3K, pAKT, pGSK3BS9, and P70S6 through RAPTOR are all lower in the hippocampus, and only PTEN is higher (and GSK3B in females).

In Dp10 females, most components of the MTOR pathway were increased in the cerebellum, but not perturbed in the hippocampus, relative to female controls, and this is reflected in the magnitudes of the bar graphs in Fig. 5d. Conversely, in male Dp10, perturbations were largely seen as increased levels in the hippocampus relative to controls. As a result, while the pattern in Fig. 5d looks very different from that of the male controls, it is consistent with trisomy perturbations.

Results of a similar analysis of MAPK components are shown in Additional file 4.

**Fig. 6:**
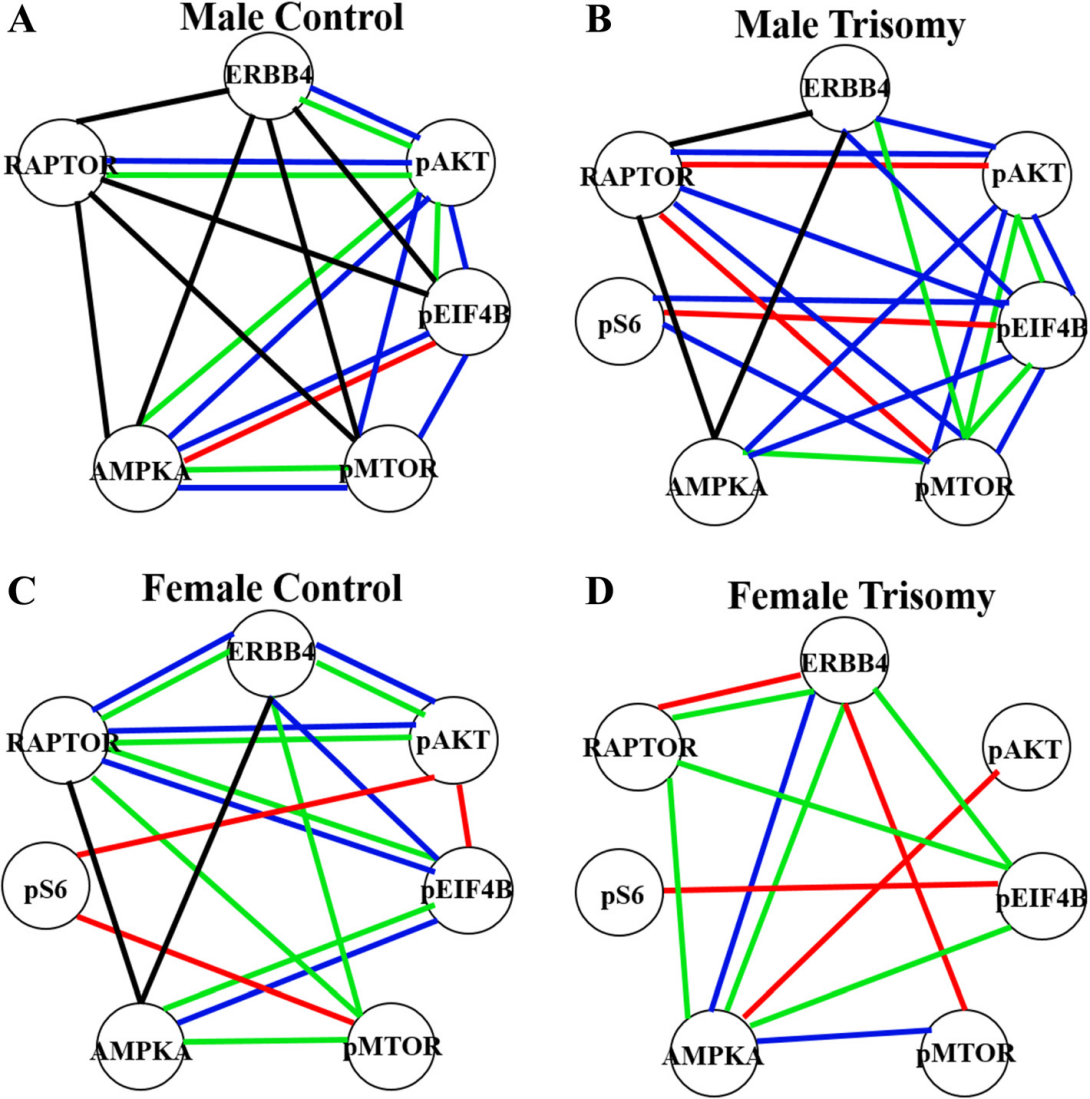
Correlation of levels of MTOR pathway components across brain regions. Correlation coefficients for proteins in each brain region and sex/genotype were determined using Spearman correlation analysis. Networks include only those protein pairs with *r* > 0.8 and *p* < 0.05, after manual inspection to exclude spurious linearities. *Red* correlations in hippocampus, *blue* cerebellum, *green* cortex, *black* all three brain regions. **a** Male controls. **b** Male Dp10. **c** Female controls. **d** Female Dp10

In prior work, correlations among functionally related proteins were noted in the hippocampus of Tc1 and Ts65Dn mice [48, 49]. In Fig. 6, we show patterns of correlations among components of the MTOR pathway in all three brain regions of the four genotype/sex groups of mice. The strongest patterns of correlations are seen in male control mice, where levels of RAPTOR, ERBB4, AMPKA, pMTOR, and pEIF4B are correlated in all three brain regions, and levels of pAKT are correlated in the cerebellum and cortex (Fig. 6a). In female controls, most of these correlations are not present in the hippocampus (Fig. 6c). In the Dp10, correlations in male mice are largely preserved in the cerebellum, but variously lost in both the hippocampus and cortex. In female Dp10, most of the correlations seen in controls in the cerebellum are lost, leaving only levels of ERBB4 and AMPKA correlated, likely as a consequence of the perturbation of protein levels in this brain region. In contrast, Dp10-specific correlations among the proteins have appeared in the hippocampus. The biological significance of the presence, and absence, of correlations is not obvious, but overall, their sexual dimorphism is consistent with dimorphic levels of protein expression.

## Discussion

Levels of ~100 proteins/protein modifications were measured in the hippocampus, cerebellum, and cortex of cohorts of adult female and male mice. Cohorts included mice trisomic for the Mmu10 region syntenic with Hsa21 and their littermate controls. Analysis uncovered both sex and genotype, brain region-specific differences in protein expression. In control mice, levels of half the proteins differed between females and males in at least one brain region, and in the hippocampus alone, expression levels of 41 proteins were significantly higher in females than those in males. Levels of only nine proteins showed sex differences in the cerebellum in controls. Trisomy differentially affected protein levels in females and males. In female trisomic mice, levels of a total of 69 proteins differed from female controls; 62 were increased in the cerebellum, while only 18 were perturbed in the hippocampus. In contrast, in male Dp10 mice, while levels of a total of 68 proteins were perturbed in at least one brain region, 49 were elevated in the hippocampus and 21 were altered in the cerebellum; the majority decreased relative to male controls. In both control and trisomic mice, cortex showed the fewest sex differences: levels of only one protein differed in control mice, and 13 and 8 were altered in female and male Dp10, respectively. Because of the sex specificities of the trisomy-induced perturbations, male and female Dp10 mice differed in levels of 15 proteins in hippocampus, 70 in the cerebellum, and 23 in the cortex, a very different profile from their littermate controls.

### Diversity of sex differences

The number of sexually dimorphic proteins is of interest given that the entire protein set was selected from those shown by mutational analysis to function in brain development, ID or LM, to be components of pathways relevant to these processes or to show abnormalities in brains of patients or mouse models of DS or AD [18, 47]. Indeed, sexually dimorphic proteins in the hippocampus of control mice include five ID proteins or their phosphorylated forms (DYRK1A, pRSK, GFAP, GLUR3, and SHH) and an additional 14 mouse LM proteins and those in trisomic mice include four ID (NR1, NR2B, pBRAF, and pFMRP) and five LM proteins. While the consequences of a mutation that alters or eliminates the function of an ID or LM protein likely will be different from a simple change in protein level, when so many learning/memory proteins differ in levels, the consequences could be significant because they impact so many downstream processes.

In both controls and trisomic mice, proteins showing sex differences are diverse in their functional classifications. They include subsets of the components of the MTOR, MAPK, and apoptosis pathways, in control mice, several AMPA receptor subunits, and in trisomic mice, NMDA receptor subunits. The cerebellum of trisomic mice shows the most dramatic sex differences, with the magnitudes of the differences, averaging >30 %, greater than in those in other genotype/brain regions. These are due to the sensitivity of female mice to trisomy of the Dp10 segment, where 62 proteins differ from control females, compared to only 21 in male Dp10.

Sex differences were also seen in levels of proteins with previously reported abnormalities in brains of patients with AD or mouse models [47]. Ten of these proteins were significantly higher in the hippocampus of control females than males. Because the majority of the original mouse experiments used only males, for some of these 26 proteins, interpretations regarding the significance of observed increases and decreases may need to be revisited after analysis of female mice is carried out. In this vein, it is of interest to note that, among proteins elevated in female controls is the amyloid precursor protein, APP, that has been shown to cause AD in families carrying a genomic duplication of the gene. Understanding the causes and consequences of naturally elevated levels in females may aid in understanding the role of elevated APP, not only in familial AD cases, but also in DS.

### Comparison with sex differences in mRNA expression

A comprehensive study of sex differences in gene expression at the RNA level found that 14 % of mRNAs expressed in the brain differed in levels between females and males [15]. This contrasts with the 40–50 % differences in protein expression identified here. This is not unreasonable because the oligonucleotide arrays are an unbiased screening of “all” transcripts, while proteins here represent a very biased selection, and also include 31 with specific post-translational modifications. However, while transcripts encoding many RPPA proteins were detected in [15], there were no overlaps between RNAs showing sex differences and the proteins identified here. Because the whole brain was used in the RNA study, many sex differences specific to the hippocampus, cerebellum, or cortex, such as observed here, would be missed, and indeed, the authors concluded that 14 % was probably a low estimate [15]. In a more recent paper, mRNA levels of 27 “mood-related” genes were examined in the frontal cortex of mice exposed to chronic mild stress [51]. Genes included several involved in GABA, serotonin, and dopamine signaling, and among them were APP, CDK5, BDNF, and AKT that were measured here. Sex differences were seen but because the mice were examined only after exposure to chromic stress [51], comparisons with data from naïve mice here are not meaningful. Lastly, because numerous regulatory mechanisms govern post-transcriptional, translational, and post-translational processes, relative RNA levels do not reliably predict differences in protein levels [52, 53].

### Comparison with sex differences in mutant phenotypes

Several proteins assayed here have been shown individually to contribute to sex-specific phenotypic features, at least when mutated. For example, in an AD-related study, when a FYN kinase null mutant was crossed with the AD “triple transgenic” model (a mouse expressing mutated forms of APP, Tau, and PSEN1), male offspring were delayed in the development of Aβ pathology and spatial learning deficits, while female offspring showed no such temporal protection, i.e., decreased levels of FYN were protective only in males [54]. Another study showed that male mice were affected by the knockout of the AMPAR subunit, GLUR1, as measured by impaired retention in CFC, while female mice were unaffected [55]. In mice deficient for the transcription factor CREB, females were more negatively affected than males, showing impairment in an easier LM task and at an earlier age than males [56]. In a knockdown of NR1, male mice showed impaired working memory in the Y maze as early as 6 weeks of age, while female mice remained unimpaired at 12 weeks [57]. Each of these proteins or paralogs showed sex differences here in controls (FYN, GLUR2-4, NR1, NR2B) or showed sex differences in response to trisomy (FYN, GLUR2-4, CREB, NR1, NR2A, NR2B). Although the differences were generally ~10–50 %, and often were increases, and not the complete or heterozygous knockout, their consequences for sex differences in the molecular pathways subserving normal learning and memory, and how these are perturbed in DS, require further investigation.

### Relevance to drug responses

Sex differences are also relevant to molecular responses to drug treatments. The effects of fluoxetine were examined in rats that had been exposed to chronic stress. When initially evaluated in the forced swim test, stressed male rats showed increased immobility while stressed females showed increased hyperactivity. In both sexes, stress response behavior was normalized by fluoxetine [58]. Levels of ERK, p38, and JNK and their phosphorylated forms were measured in cytosolic and nuclear fractions of the hippocampus. Complex and sex-specific changes in levels and subcellular distributions of these proteins occurred in response to stress and to fluoxetine [58, 59]. So, while behavioral outcomes appeared to be the same in male and female animals, the molecular pathways to achieving this common result clearly differed between sexes. Levels of these same proteins differed here between sexes in control mice and in their perturbations in trisomy. Fluoxetine is of interest in DS because it has been shown to rescue LM deficits in another mouse model of DS, the Ts65Dn [60–62]. Two points require consideration. First, the Ts65Dn are trisomic for a completely different set of Hsa21 orthologs than are the Dp10 mice. Thus, in full trisomy Hsa21 (i.e., >95 % of individuals with DS), the responses to fluoxetine, at least at the molecular level, will most likely be different from those in the Ts65Dn because of influences of the Mmu10 orthologs. If, or how, this would affect behavioral outcomes is a complete unknown. Second, while male and female mice were used in the Ts65Dn fluoxetine experiments, and efficacy was reported to show no sex differences, the number of animals per group was typically small (often a total, males plus females, of only four to six individuals), so that significant sex differences would be difficult to detect. Given our limited knowledge of sex differences at the molecular level, and in drug responses, it is premature to pool sexes in data analysis, especially in proposing clinical trials for cognition in ID. No experimental data are available in any DS mouse model regarding sex differences in molecular responses to any drug currently in or proposed for clinical trials [63]; these responses may differ, not only in full trisomy vs. in different partial trisomy mouse models but also in females vs. males.

### Molecular contributions to sex and genotype differences

**Fig. 7:**
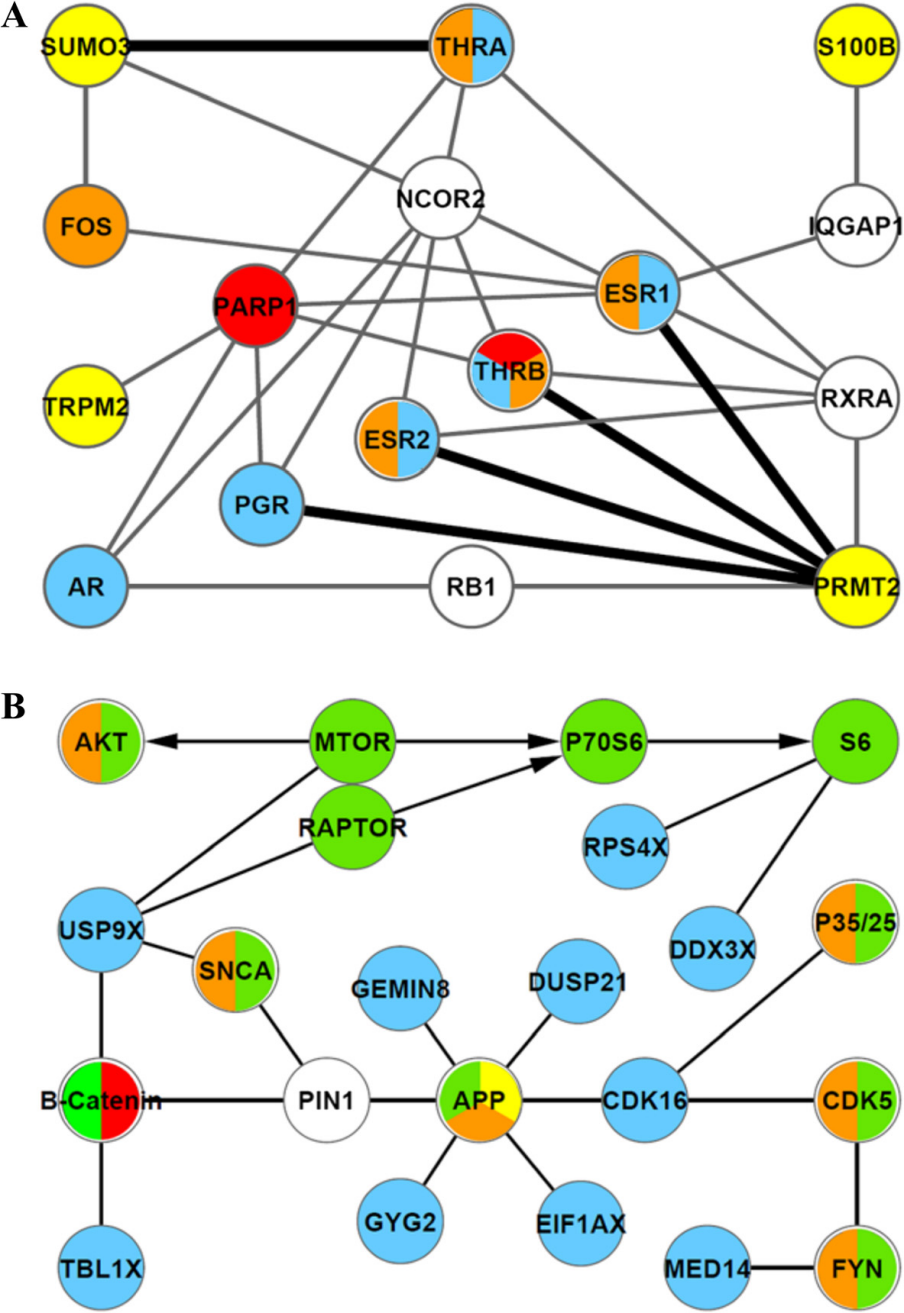
Protein interaction networks. Protein interactions, retrieved from curated public databases, are indicated by *lines* connecting two nodes. Nodes are color-coded: *yellow* Hsa21-encoded protein, *red* human ID protein [18], *orange* mouse LM protein (The Mammalian Phenotype Database). **a** Interactions between Hsa21 proteins and sex or thyroid hormone receptors (*blue*); *heavy lines* direct interactions with a Dp10 protein. **b** Interactions of RPPA proteins (*green*) that showed an abnormal level in at least one brain region/sex/genotype with X chromosome-encoded proteins (*blue*) that escape silencing by X inactivation [10–12]. *Arrows* indicate activation in the MTOR pathway

Sex hormones, X chromosome genes that escape inactivation, and environmental effects all may contribute to the sexually dimorphic patterns of protein expression observed here in control mice. That these patterns are different in Dp10 mice suggests that trisomic Mmu10 genes impact one or more of these normal processes. Based on the known functions of Mmu10 trisomic genes, candidates for perturbations of sex differences in expression can be proposed (although we note that levels of gonadal hormones were not measured). Figure 7a shows a network of protein interactions connecting Dp10 trisomic genes with estrogen, progesterone, androgen, and thyroid hormone receptors. The protein methyltransferase, PRMT2, is of particular interest because it directly modifies and activates ESR1, ESR2, PGR, and THRB and indirectly affects AR [41, 42]. In the cerebellum, levels of PRMT2 in female controls were 25 % lower than those in male controls; trisomy served to increase PRMT2 levels in females by >90 % and decrease them in males by >30 %. Functional consequences for the cerebellum in DS are of interest because of well-documented abnormalities in DS regarding volume and cell numbers [64, 65]. Cerebellar function is relevant, not only to motor control but also to higher cognitive functions related to language and executive function [66, 67]. The Hsa21-encoded small ubiquitin-like protein, SUMO3, modifies the nuclear receptor co-repressor, NCOR2 which in turn inhibits the activities of ESR1, ESR2, PGR, and AR [42, 68]. Levels of SUMO3 were not measured here, but where both SUMO3 and PRMT2 are overexpressed, the consequences for regulation of activity levels of the hormone receptors will further complicate predictions for phenotypic features in DS. Information on the Dp10 regarding cellular/structural cerebellar abnormalities is currently lacking.

Knockouts of two genes trisomic in the Dp10 have been shown to be associated with sex-specific phenotypes. TRPM2, a calcium-permeable cation channel primarily activated by intracellular adenosine-diphosphate ribose (ADPR) [69, 70], shows enhanced activation with exposure to hydrogen peroxide and elevated oxidative stress and leads to cell death. Knockout of the TPRM2 protects male, but not female, mice from effects of ischemia, and interaction with the AR through PARP1 has been proposed as the mechanism [71, 72]. Elevated levels of oxidative stress have been well documented in DS [73], implicating a contribution from TRPM2 overexpression. In addition, hyperactivation of TRPM2 channels has recently been implicated as a downstream consequence of Aβ increases in the AD brain and as a mechanism contributing to cerebrovascular pathologies in AD [74]. These latter experiments were conducted only in male mice. Possible sex differences in these features in DS need to be considered.

Not included in Fig. 7a but also showing sex differences is the adenosine deaminase, ADAR2, that edits pre-mRNAs encoding several glutamate receptor subunits and a serotonin and a GABAA receptor subunit [31–33]. Editing alters the amino acid sequence in these substrates, consequently modulating receptor functional properties and activity levels. Knockout of ADAR2 in control mice was shown to impair hearing and the acoustic startle response in male mice but leave females unaffected [34]. The molecular mechanisms producing this phenotype are not known. ADAR2 levels were elevated by 50 % in the hippocampus of male, but not female Dp10 mice.

In humans, although it has been consistently shown that ~15 % of X chromosome genes escape inactivation, the consequences for protein levels have not been well characterized. The number and identity of the genes expressed from both X chromosomes, and the resulting mRNA levels, varies among individuals and between tissues/cell types [10–15, 75–78]. In mouse, fewer X chromosome genes, ~3–7 %, have been reported to escape inactivation [79]. It is not possible to generalize the consequences for sex differences in the total proteome or to extend observations in mouse to human; however, some possibilities are illustrated in Fig. 7b. The network includes X chromosome-encoded proteins that escape inactivation in humans and interact with APP and components of the MTOR pathway, each of which showed sex differences here in control mice or perturbation in trisomy. This would predict a molecular contribution to sex differences in protein expression in humans, with the potential for novel sex-specific differences in DS, features that can be explored in future experiments.

Environmental conditions can also influence gene expression, potentially contributing to sex or genotype differences. General environmental conditions are controlled, e.g., all mice are exposed to the same level and frequency of noise, light/dark cycle, and access to food and water. In addition, all mice were sacrificed between noon and 2 p.m. to control for normal circadian variations in protein expression. However, if there are sex or genotype differences in sensitivity to any of these features, some (generally unpredictable) part of the proteome would be affected. Behavioral studies are necessary to determine if the Dp10, males or females, are differentially affected by environmental conditions of noise or handling or if they exhibit altered sleep/wake or feeding patterns.

An additional environmental influence on the proteome arises from housing conditions and potential effects of social hierarchy. A dominant male would have higher levels of androgens than a submissive male, with downstream consequences for some protein expression. All mice here were housed with their littermates of the same sex. Because we did not ascertain the effects of trisomy on the propensity for dominance, there are three possible scenarios: (i) control and Dp10 males are equally likely to be the dominant animal, (ii) controls will always be dominant over a Dp10, or (iii) a Dp10 will always be dominant over a control. Given the number of each genotype in each of the seven litters (Additional file 1), at most six control mice or five Dp10 mice could be dominant in any scenario. In test calculations, we assumed that dominance results in a 50 % increase (direct or indirect) in the level of protein A; in no case could this produce a difference between controls and trisomy that is significant after correction for multiple testing (data not shown). We therefore conclude that none of the differences we report is falsely attributed to genotype instead of dominance. However, by the same calculations, a 50 % difference that is due to dominance could be sufficient to mask a true genotype difference of the magnitudes observed here, if the genotype difference is opposite in direction (data not shown). By this reasoning therefore, it is possible that there are actually more proteins perturbed in trisomy than we detected. To our knowledge, there have been no comprehensive protein profiles generated from dominant vs. submissive mice. In future experiments where social hierarchy is considered, it will be of interest to ascertain the nature and magnitude of effects on the protein set queried here.

### Implications for sex differences in DS

While sex differences in the typical population in cognitive strengths, neuropsychiatric disease incidence and severity, and drug responses have been well documented, little is known about the molecular correlates of the differences. Sex differences in the same features in people with DS, and whether they simply reflect those in the typical population, have not been commonly reported [80]. However, there are reports to suggest that this should be explored. For example, examination of the records of >1300 individuals with DS spanning 1953–2000 found that life span for females was significantly shorter than for males (<58 vs. >61 years, respectively) [81]. This is opposite to the typical population and to a population with ID not due to DS. There is evidence that sex differences in cognitive profiles exist in DS. In assessments carried out over a span of 7 years of a cohort of people with DS with similar mean IQ levels (~40), females outperformed males on a subset of tests from the Wechsler Intelligence Scale [82]. This observation is supported and extended in results from a recent comprehensive analysis of adults with DS; women performed significantly better than men on several evaluations including those for memory and executive function [83]. A challenge in many cellular/molecular studies is small sample size. For example, the intriguing results characterizing cerebellar deficits in cell proliferation [84] examined only three females and four males, too few for reliable detection or exclusion of sex differences. A few studies using the Ts65Dn mouse model of DS have shown sex differences. Female Ts65Dn were shown to have lower numbers of the basal forebrain cholinergic neurons [85], and sex differences in anxiety after exposure to predators have been reported [86]. These examples obviously are neither comprehensive nor even very extensive. It should not be assumed, therefore, that sex differences in DS, in particular in cognitive performance, and in mechanisms to cope with stress and anxiety that can impact cognitive performance, are insignificant DS or not different from the typical population, until a concerted effort to identify them is carried out.

## Conclusions

The number and nature of significant perturbations in protein expression in 8-month-old Dp10 mice contrasts with the report of no LM deficits in 2–4-month-old (male) Dp10 [23]. These disparate observations could be explained if the protein abnormalities are age-dependent and absent in younger mice. If this is true, it will be of interest to determine if the protein abnormalities at 8 months also reflect an age-dependent development of LM deficits. Experiments to address these possibilities are in progress. If, however, these protein abnormalities are not associated with LM deficits, an alternate explanation is that one or more trisomic proteins directly or indirectly act to protect the Dp10 from the deleterious effects of overexpression of other trisomic genes, i.e., the constellation of molecular abnormalities seen here is a sum of deleterious effects and neutralizing, compensatory responses. This scenario would have consequences for full trisomy Hsa21, where trisomy of Mmu10 orthologs could influence the DS phenotype and responses to drug treatments. It remains possible, of course, that none of the products of the Dp10 trisomic genes, individually or collectively, or the observed downstream abnormalities, negatively perturbs neurological function. This would, however, be surprising, given the known roles of many of these proteins in molecular and cellular processes underlying normal LM and ID. The involvement of Hsa21 proteins shown in Fig. 7, i.e., PRMT2, SUMO3, S100B, TRPM2, in modulating activities of thyroid, estrogen, and other sex hormone receptors, and in interactions with many ID proteins certainly suggests sex differences may well exist in DS, with the consequence that sex differences in drug responses could also be significant. Further experiments are clearly necessary to compare not only protein expression consequences but also behavioral and drug responses of the Mmu10 trisomic region in the presence of trisomy of the Mmu16 and Mmu17 regions. While the extents of the sex differences in controls and Dp10 mice may be surprising, they are entirely consistent with and supportive of past [87] and more recent calls [88, 89] for inclusion of females in both cell and animal studies in general and preclinical evaluations of drug treatments in particular. It is important to determine how results here in mice extend to humans.

## Additional files

Additional file 1: Table S1. Mouse information: age, sex, littermates, and sample weights. (XLS 40 kb) Additional file 2: Table S2. Protein and antibody information: source, catalog number, dilution factor, and protein classification. (XLSX 778 kb) Additional file 3: Table S3. Results of all pairwise comparisons: all proteins in each brain region. (XLS 168 kb) Additional file 4: Figures S1–S4.
**Figure S1.** Sex and genotype differences for TIAM1, CBS, and RRP1 in the cortex of the Dp10. **Figure S2.** Sex and genotype differences for MAPK pathway in the hippocampus of the Dp10. **Figure S3A, 3B.** Sex and genotype differences for AD-related proteins in the hippocampus (A) and cerebellum (B) of the Dp10. **Figure S4.** Sex and genotype differences for the MAPK pathway comparisons between the cerebellum vs. hippocampus. (PPTX 393 kb)

## Abbreviations

AD: Alzheimer’s disease
Dp10: Dp(10)1Yey
DS: Down syndrome
ID: intellectual disability
LM: learning/memory
Mmu: mouse chromosome
RPPA: reverse phase protein array
