# Supplementary material for: Sex differences in protein expression in the mouse brain and their perturbations in a model of Down syndrome
Source: Biol Sex Differ. 2015 Nov 9;6:24. doi: 10.1186/s13293-015-0043-9 (PMC4640233; doi:10.1186/s13293-015-0043-9)
Supplement: Additional file 4: Figures S1–S4. — Figure S1. Sex and genotype differences for TIAM1, CBS, and RRP1 in the cortex of the Dp10. Figure S2. Sex and genotype differences for MAPK pathway in the hippocampus of the Dp10. Figure S3A, 3B. Sex and genotype differences for AD-related proteins in the hippocampus (A) and cerebellum (B) of the Dp10. Figure S4. Sex and genotype differences for the MAPK pathway comparisons between the cerebellum vs. hippocampus. (PPTX 393 kb) [file 13293_2015_43_MOESM4_ESM.pptx]

## Slide 1
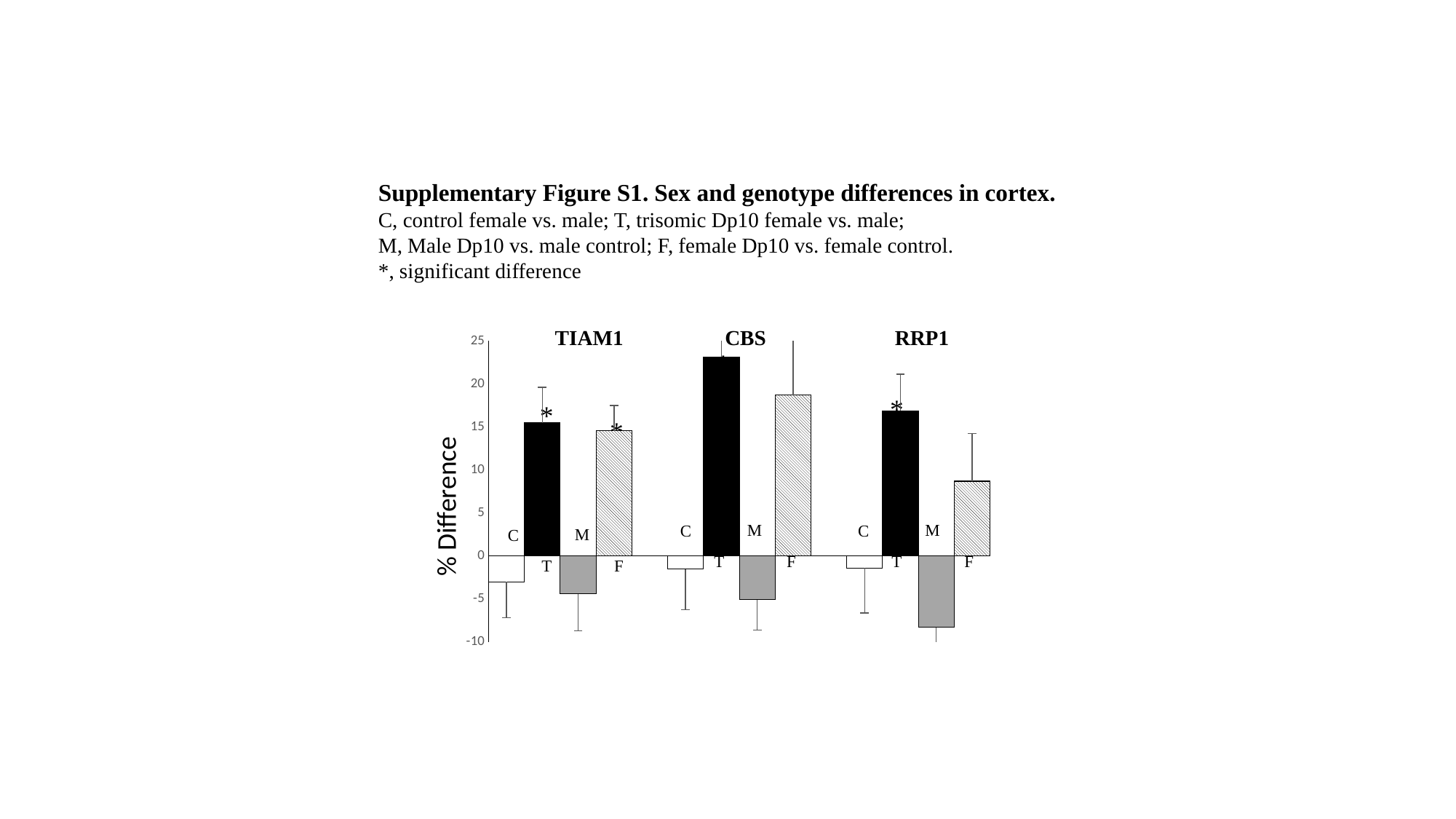

Supplementary Figure S1. Sex and genotype differences in cortex.
C, control female vs. male; T, trisomic Dp10 female vs. male;
M, Male Dp10 vs. male control; F, female Dp10 vs. female control.
*, significant difference
RRP1
TIAM1
CBS
### Chart
| Category | |
|---|---|% Difference
*
*
*
*
M
M
C
C
M
C
T
F
T
F
T
F

## Slide 2
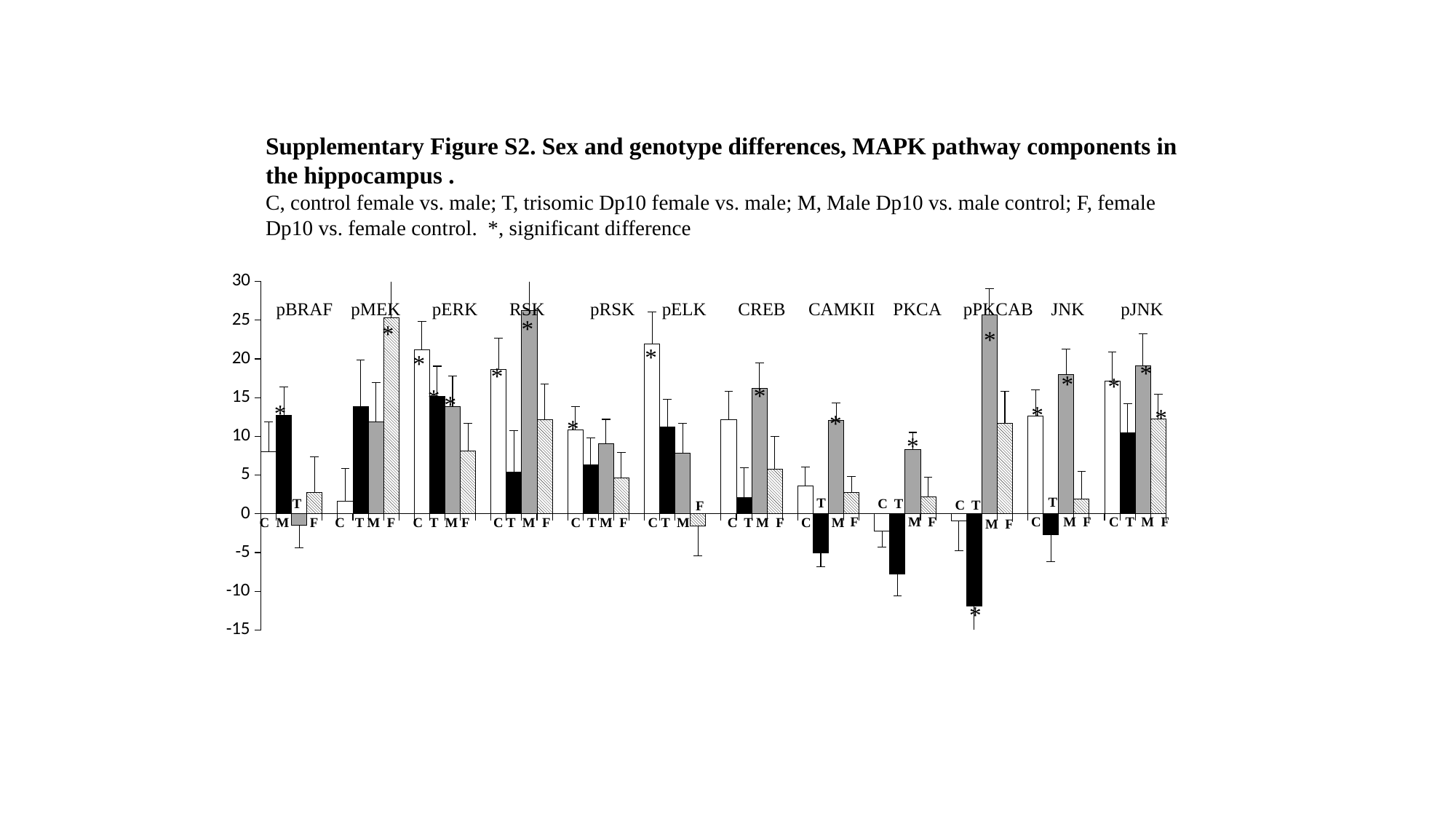

Supplementary Figure S2. Sex and genotype differences, MAPK pathway components in the hippocampus .
C, control female vs. male; T, trisomic Dp10 female vs. male; M, Male Dp10 vs. male control; F, female Dp10 vs. female control. *, significant difference
### Chart
| Category | |
|---|---|pBRAF pMEK pERK RSK pRSK pELK CREB CAMKII PKCA pPKCAB JNK pJNK
*
*
*
*
*
*
*
*
*
*
*
*
*
*
*
*
*
*
T
T
T
C T
C T
F
F
M F
C
M F
C T M F
C M F C T M F
C T M F C T M F
 C T M F C T M
 C T M F C M
M F
*

## Slide 3
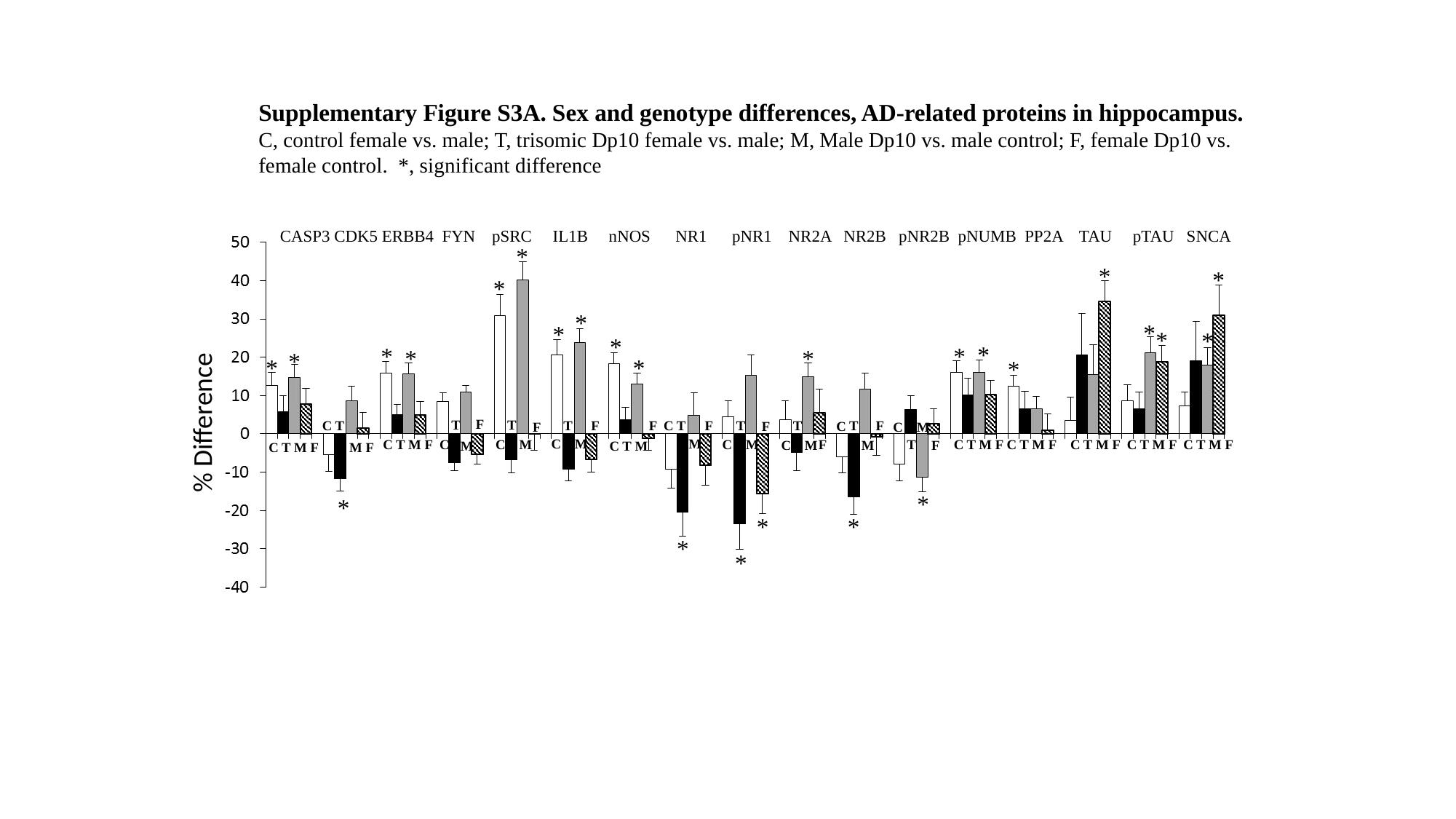

Supplementary Figure S3A. Sex and genotype differences, AD-related proteins in hippocampus.
C, control female vs. male; T, trisomic Dp10 female vs. male; M, Male Dp10 vs. male control; F, female Dp10 vs. female control. *, significant difference
CASP3 CDK5 ERBB4 FYN pSRC IL1B nNOS NR1 pNR1 NR2A NR2B pNR2B pNUMB PP2A TAU pTAU SNCA
*
*
*
*
*
*
*
*
*
*
*
*
*
*
*
*
*
*
*
F
T
T
T
F C T
T
T
F
C T
F
F
T
C
F
C M
F
M
C M
C T M F C T M F C T M F
T
C T M F C
C T M F C T M F
C M
C M
F
F
C M
M
C T M
M
C T M F M F
*
*
*
*
*
*
% Difference

## Slide 4
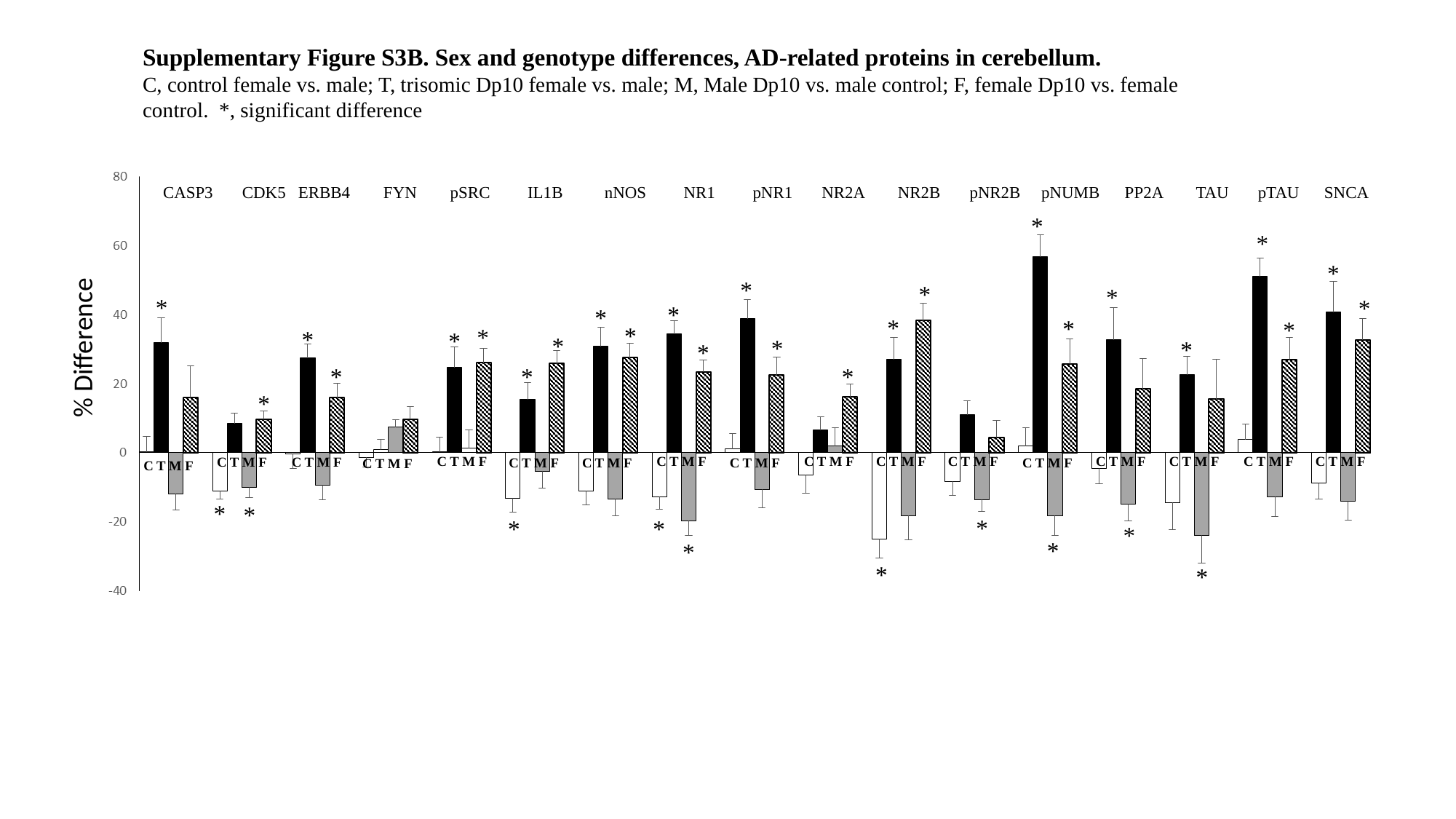

Supplementary Figure S3B. Sex and genotype differences, AD-related proteins in cerebellum.
C, control female vs. male; T, trisomic Dp10 female vs. male; M, Male Dp10 vs. male control; F, female Dp10 vs. female control. *, significant difference
CASP3 CDK5 ERBB4 FYN pSRC IL1B nNOS NR1 pNR1 NR2A NR2B pNR2B pNUMB PP2A TAU pTAU SNCA
*
*
*
*
*
*
*
*
*
*
*
*
*
*
*
*
*
*
*
*
*
*
*
*
*
C T M F
C T M F
C T M F
C T M F
C T M F
C T M F
C T M F
C T M F
C T M F
C T M F
C T M F
C T M F
C T M F
C T M F
C T M F
C T M F
C T M F
*
*
*
*
*
*
*
*
*
*
% Difference

## Slide 5
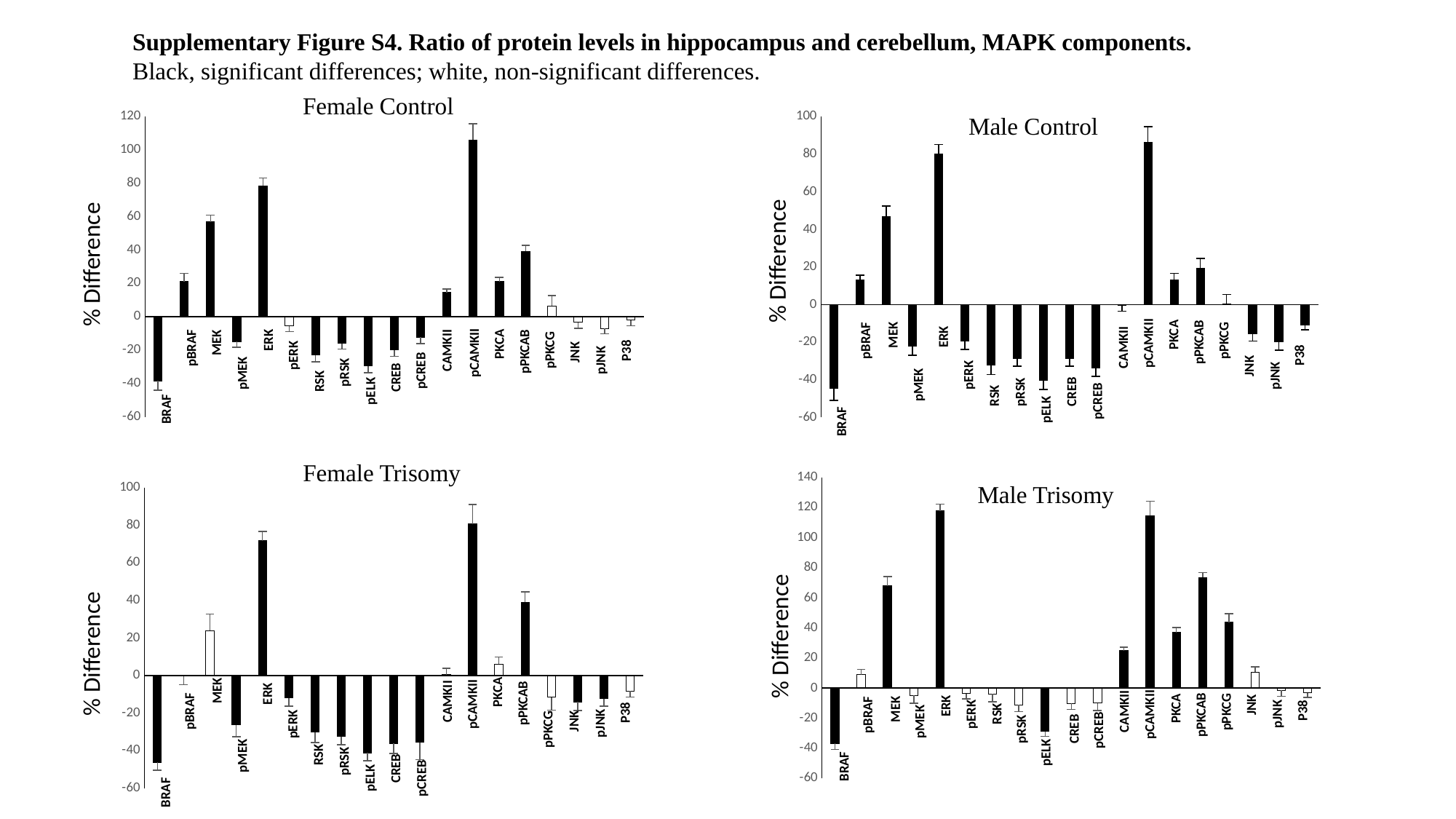

Supplementary Figure S4. Ratio of protein levels in hippocampus and cerebellum, MAPK components.
Black, significant differences; white, non-significant differences.
Female Control
### Chart
| Category | |
|---|---|ERK
MEK
PKCA
pBRAF
pPKCG
P38
CAMKII
pPKCAB
JNK
pCAMKII
pERK
pJNK
pCREB
pRSK
pMEK
CREB
RSK
pELK
BRAF
### Chart
| Category | |
|---|---|MEK
PKCA
ERK
pBRAF
pPKCG
pPKCAB
pCAMKII
CAMKII
P38
JNK
pERK
pJNK
pMEK
CREB
pRSK
RSK
pCREB
pELK
BRAF
Male Control
% Difference
% Difference
Female Trisomy
### Chart
| Category | |
|---|---|JNK
ERK
PKCA
MEK
P38
CAMKII
pPKCG
RSK
pJNK
pERK
pCAMKII
pBRAF
pPKCAB
pMEK
CREB
pRSK
pCREB
pELK
BRAF
Male Trisomy
### Chart
| Category | |
|---|---|MEK
PKCA
ERK
CAMKII
pPKCAB
pCAMKII
pBRAF
P38
JNK
pJNK
pERK
pPKCG
RSK
pMEK
pRSK
CREB
pELK
pCREB
BRAF
% Difference
% Difference
